# IL-6, malnutrition, and short-term mortality in prevalent hemodialysis patients

**DOI:** 10.1590/2175-8239-JBN-2022-0151en

**Published:** 2022-12-05

**Authors:** Marisa Roldão, Rachele Escoli, Paulo Santos

**Affiliations:** 1 Centro Hospitalar do Médio Tejo, Departamento de Nefrologia, Torres Novas, Portugal.

Dear Editor,

Inflammation has been associated with increased mortality in hemodialysis (HD) patients^1,2,3,4^. Some previous studies had suggested an association between higher interleukin (IL)-6 levels and mortality risk in prevalent HD patients. The influence of serum IL-6 levels on nutritional status remains, however, to be elucidated. The aim of this study was to evaluate the association between serum IL-6 levels and nutritional status, and its impact on short-term mortality in prevalent HD patients.

We conducted a retrospective single-center study of prevalent HD patients between January 2020 and January 2021. Patients with acute or chronic inflammatory or infectious disease and active malignancy were excluded. According to the mean IL-6 level measured at baseline, patients were categorized into two cohorts: IL-6 ≤ 17pg/mL and IL-6 > 17pg/mL. Baseline clinical and demographic data were collected. Laboratory parameters were measured before the second HD session of the week, including hemoglobin (Hgb), ferritin, serum iron, transferrin saturation index (TSI), albumin, C-reactive protein (CRP), phosphorus (P^+^), and calcium (Ca^2+^). PTH was measured by electrochemiluminescence and IL-6 levels were measured by ELISA. Baseline characteristics and laboratory data were compared among groups using the Student’s t-test for normally distributed continuous variables, Mann-Whitney U-test for skewed distributed continuous variables, and Chi-square test for categorical variables. One-year all-cause mortality was assessed using standard survival methods. Statistical analysis was performed using SPSS (Version 23 for Mac OSX).

The mean age of the included 61 prevalent HD patients was 74.96 ± 12.89 years, 34 (55.7%) were male, and 25 (41%) were diabetic. Mean IL-6 level was 17.3 ± 28.19 pg/mL. Fourteen patients (23%) had IL-6 > 17pg/mL. There was no gender, comorbidity, HD vintage, or modality difference among groups. Patients with IL-6 > 17pg/mL were older (80.50 ± 7.82 vs 73.13 ± 12.71 years, *p* = 0.045), had higher c-reactive protein (CRP) (3.81 ± 4.48 vs 0.67 ± 0.51 mg/dL, *p* = 0.001), lower serum iron (37.80 ± 18.36 vs 59.57 ± 34.44, *p* = 0.014), and lower transferrin saturation index (TSI) (19.36 ± 8.63 vs 31.29 ± 23.74%, *p* = 0.046). Those patients had also higher serum ferritin levels, although not statistically significant (448.43 ± 307.79 mg/dL vs 332.98 ± 248.12 mg/dL, *p* = 0.059). Regarding nutritional parameters, patients with IL-6 > 17 pg/mL had lower albumin (3.30 ± 0.45 vs 3.69 ± 0.39g/dL, *p* = 0.003), lean mass index (LMI) (10.22 ± 2.07 vs 12.65 ± 4.44 kg/m^2^, *p* = 0.014), body mass index (BMI) (26 ± 6.71 vs 24.25 ± 5.26 kg/m^2^, *p* = 0.666), and cholesterol (157.64 ± 52.37 vs 170.96 ± 50.34 mg/dL, *p* = 0.307), but without statistical significance. During the study follow-up period, 9 (14.8%) patients died. One-year survival was 91.4% in the group with IL-6 ≤ 17 pg/mL and 64.3% in those with IL-6 > 17 pg/mL (log-rank = 5.89; *p* = 0.015). Using a Cox proportional hazards analysis, we observed that patients with IL-6 > 17 pg/mL had an increased risk of all-cause mortality at one year compared to those with IL-6 ≤ 17 pg/mL (HR: 4.43; 95% CI: 1.18-16.51; *p* = 0.027).

Our results suggest that higher serum IL-6 levels in prevalent HD patients are associated with markers of malnutrition, such as lower albumin, LMI, BMI, and cholesterol, and increased risk of all-cause short-term mortality. Our results corroborate previous studies that reported inflammation, protein-energy wasting (PEW), and cardiovascular morbidity to be interrelated in HD patients, each additionally contributing to higher mortality in prevalent HD patients.
